# Positive blood cultures in a patient recovering from febrile neutropenia

**DOI:** 10.1099/jmmcr.0.005038

**Published:** 2016-06-28

**Authors:** Aleksandra Stefanovic, Alissa Wright, Vincent Tang, Linda Hoang

**Affiliations:** ^1^​ Division of Medical Microbiology, Department of Pathology and Laboratory Medicine, Vancouver General Hospital, Vancouver, BC, Canada; ^2^​ Division of Infectious Diseases, Department of Internal Medicine, Vancouver General Hospital, Vancouver, BC, Canada; ^3^​ BC Public Health Microbiology and Reference Laboratory, Vancouver, BC, Canada

**Keywords:** *Scedosporium prolificans*, Febrile neutropenia, Immunosuppressed host, Fungemia

## Case summary

A 44-year-old male returned from India where he was hospitalized and diagnosed with Hemophagocytic Lymphohistiocytosis (HLH). He had been initiated on dexamethasone followed by a prednisone taper. On return to Canada, he presented with non-productive cough, fevers and pancytopenia and was admitted to the Intensive Care Unit (ICU) with sepsis. He underwent a bone marrow biopsy and the diagnosis of intravascular large B cell lymphoma with secondary HLH was made. He was started on a regimen of cyclophosphamide, doxorubicin, rituximab and etoposide along with filgrastim (G-CSF). He subsequently developed febrile neutropenia and his blood cultures initially grew OXA-48 carbapenemase-producing *Klebsiella pneumoniae*, Extended Spectrum β- Lactamase (ESBL)-producing *Escherichia coli* and *Streptococcus gordonii*. He received combination therapy with high-dose IV imipenem-cilastin, IV piperacillin-tazobactam, IV colistin, IV tigecycline, and high-dose oral fosfomycin.

**Question**

**Fig. 1.**
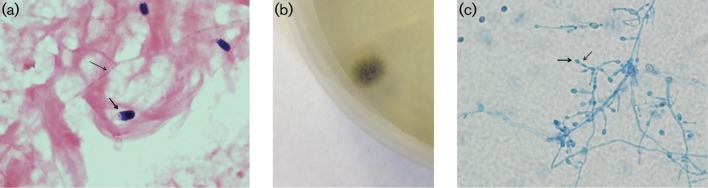
(a) Gram stain of blood culture showing septate hyphae (thin arrow) and ovoid conidia with truncated base (thick arrow); magnification ×1000. (b) Growth of *Scedosporium prolificans* on Sabaroud Dextrose Agar (SDA) at 30 °C for 4 days; surface of the colony. (c) Lactophenol blue stain of the fungus colony showing septate hyphea with conidiophores having swollen base and tapered ‘neck’ (thick red arrow) with conidia (thin red arrow); magnification ×400.

After 10 days of continuous bacteraemia with OXA-48 *Klebsiella pneumoniae*, blood cultures showed a new organism on Gram stain (Fig. 1). What is your diagnosis?

**Answer options**

1. Candidaemia
2. Invasive Aspergillosis
3. Disseminated *Scedosporium* infection
4. Disseminated *Fusarium* infection

Patient had undergone bronchoalveolar lavage (BAL) for his initial cough, fevers and radiological findings of multiple lung nodules of ground glass attenuation, and right middle lobe and left lower lobe areas of consolidation on CT chest. This BAL grew *Scedosporium prolificans* and he was started on voriconazole while the isolate was sent for antifungal susceptibility testing. Antifungal susceptibility testing was performed at Reference Mycology Laboratory (Reference Mycology, University of Alberta, Edmonton, Canada) and demonstrated MICs off-scale for most antifungal agents tested (amphotericin B, itraconazole, micafungin, posaconazole, 5-Flucytosine) and a voriconazole MIC of 16. Despite being on voriconazole he developed fungemia with *Scedosporium prolificans* after the blood cultures were cleared of OXA-48 *K. pneumonia* bacteraemia. Initially, blood culture with this fungus was reported as growth of yeast and micafungin was added to his regimen. However, on further review it was determined that there were septate hyphae on the Gram stain from the blood culture along with ovoid conidia with a truncated base (see Fig. 1a). Growth of olive green colonies was noted on SAB Dextrose Agar (Fig. 1b) after day 4 of culture. Lactophenol blue staining of colony growth demonstrated septate hyaline hyphae with conidiophores having swollen base and tapered neck, ie ‘flask-shape’ with oval conidia (Fig. 1c), which is characteristic of *Scedosporium prolificans*.

## Discussion

**Correct Answer: **3. Disseminated *Scedosporium* infection.

The two main species of the genus *Scedosporium* of human significance are *Scedosporium prolificans* and *Scedosporium apiospermum* (with teleomorph state of *Pseudallescheria boydii*) (Ortoneda *et al.*, 2002). Both are ubiquitous filamentous fungi found in environmental sources such as soil and decaying vegetation (Husain *et al.*, 2005). Their clinical manifestations range from colonization of respiratory tract, invasive localized disease to disseminated infections. *Scedosporium prolificans* is thought to be the more virulent species and is associated with disseminated infections in immunocompromised hosts such as patients with hematopoietic stem cell transplant and neutropenia (Ortoneda *et al.*, 2002; Husain *et al.*, 2005). Still *Scedosporium* fungemia is infrequently seen in blood cultures in clinical laboratories and the fungal conidia on blood culture Gram stain can be initially mistaken for oval yeast cells. However, the presence of truncated conidia along with septate hyphae is an important distinguishing feature from oval yeast cells with pseudohyphae. This initial distinction is important for antifungal choice as usual treatment of candidaemia differs significantly from that required in infections due to species of *Scedosporium*.

Furthermore, *Scedosporium prolificans* is the more resistant of the two species of *Scedosporium* and even though there are no validated interpretive breakpoints for determining resistance to antifungal agents, our isolate had MICs off-scale (highly resistant) to most antifungals tested and a voriconazole MIC of 16. This is comparable to other studies where a median voriconazole MIC_50_ of 4 (2–16) has been reported, a level well above the achievable free drug concentration in most patients (Cuenca-Estrella *et al.*, 1999; Carrillo & Guarro, 2001; Meletiadis *et al.*, 2002; Cortez *et al.*, 2008). This patient was started on voriconazole after the isolate was recovered from the BAL and while the resistance results were pending. Despite being on this treatment and recovery of his neutropenia, he developed disseminated infection with *Scedosporium prolificans.* Voriconazole therapeutic drug levels were checked and found to be sub-therapeutic at 0.7 (normal 1.0–5.5). The voriconazole dose was adjusted and high-dose terbinafine as well as miltefosine were added.

The European Society of Clinical Microbiology and Infectious Diseases (ESCMID) and the European Confederation of Medical Mycology (ECMM) joint guidelines advocate for treatment with voriconazole as a first line therapy for disseminated *Scedosporium prolificans* infection in immunocompromised patients (Tortorano *et al.*, 2014). They also suggest therapeutic drug monitoring (TDM) of voriconazole as levels vary among individuals due to the differences in CYP3A4 metabolism. Combination of voriconazole with terbinafine is also listed as a treatment option as there is demonstration of *in vitro* synergy; however there is still a lack of clinical data and only case reports are published on utility of this combination (Meletiadis *et al.*, 2003; Howden *et al.*, 2003; Whyte *et al.*, 2005). As our patient developed breakthrough scedosporiosis while on voriconazole, miltefosine was also added as salvage therapy based on clinical cases reports and *in vitro* data of its activity in combination with voriconazole (Kesson *et al.*, 2009; Trubiano *et al.*, 2014; Compain *et al.*, 2015). Without the recovery of normal immune function, mortality in disseminated *Scedosporium prolificans* infections has been reported to be up to 87.5 % despite antifungal treatment (Rodriguez-Tudela *et al.*, 2009). Unfortunately, our patient succumbed to a disseminated multi-drug resistant *Scedosporium prolificans* infection despite recovery of his counts and sterilization of his carbepenemase-producing bacteraemia.
